# Simulated intra-individual skin color changes and their impact on facial attractiveness across Japanese, Chinese, and Caucasian faces

**DOI:** 10.3389/fpsyg.2025.1600306

**Published:** 2025-09-05

**Authors:** Koyo Koizumi, Megumi Nagashima, Naoyasu Hirao, Hideki Ohira

**Affiliations:** ^1^MIRAI Technology Institute, Shiseido Co., Ltd., Yokohama, Japan; ^2^Department of Psychology, Graduate School of Informatics, Nagoya University, Nagoya, Japan

**Keywords:** facial attractiveness, facial perception, survey, Chinese, Japanese, Caucasian

## Abstract

**Introduction:**

Understanding the influence of facial features on attractiveness is important for the development of cosmetics that improve attractiveness. Although it is known that changes in skin color affect attractiveness, few studies have investigated whether changes in skin color within individuals affect attractiveness, or whether these effects vary across geographic and cultural contexts. Therefore, through online surveys, we aimed to investigate changes in visual attractiveness due to intra-individual changes in skin color, using images of Japanese, Chinese, and Caucasian faces.

**Method:**

Ten patterns of skin color change were applied to the average faces of Japanese, Chinese, and Caucasian men and women in their 20s. Using online surveys,1,200 Japanese, 1,518 Chinese, and 1,200 Italian participants were asked to rate the attractiveness of the facial images on a 5-point Likert scale.

**Results:**

Changes in attractiveness associated with changes in skin color were observed in all surveys. Furthermore, we found geographic, cultural, and sex differences in changes in attractiveness associated with changes in skin color.

**Conclusion:**

The results indicate that intra-individual changes in skin color can alter facial attractiveness. Furthermore, changes in attractiveness in response to skin color changes may vary according to facial sex and geographic and cultural backgrounds.

## 1 Introduction

Individuals use cosmetics to improve their appearance and attractiveness. Makeup can correct skin brightness and color (Kim et al., 2023). Skin color, a major factor that impacts attractiveness (Fink et al., 2006; Fink et al., 2012), is affected by melanin and hemoglobin levels. Skin color can change owing to increased melanin from sun exposure, oxygenation and deoxygenation of the blood, and stress (Mohiuddin, 2019; Stephen et al., 2009; Watanabe and Kageyama, 2017). Moreover, these changes can occur in a short period of time, from a few hours to a few days (Clydesdale et al., 2001; Hönigsmann et al., 1986; Leary et al., 1992). If changes in an individual’s skin color has a negative impact on attractiveness, cosmetics are an effective method to manage skin color (Kim et al., 2023). Furthermore, the impact of skin color change on attractiveness may vary according to geographic and cultural backgrounds (Caisey et al., 2006; Yamamoto et al., 2002). Understanding these variations is crucial for the development of global cosmetic products.

Various studies have investigated the relationship between skin color and facial attractiveness. Increased facial redness in Caucasian male faces increased attractiveness through improved health (Thorstenson et al., 2017). Meanwhile, dark skin was reportedly more attractive than bright skin on the faces of Caucasian females (Fink et al., 2001). Apparent health has been reported to influence facial attractiveness (Jones et al., 2016). The association between attractiveness and apparent health has been examined not only in Western but also in Japanese faces (Rhodes et al., 2007). Skin redness indicates increased blood perfusion and oxygenation and increases apparent health (Stephen et al., 2009). Melanin and carotenoids influence skin brightness and yellowness; increased yellowness and decreased brightness in Caucasian faces are known to increase apparent health (Stephen et al., 2012). There was a positive correlation between facial yellowness and health in Caucasian females (Henderson et al., 2016). Skin color can evoke the state of an individual’s blood circulation and carotenoids absorbed from fruits and vegetable consumption, which can alter one’s perceived health (Stephen et al., 2009; Stephen et al., 2012). Moreover, perceived health is correlated with attractiveness, and skin color is considered a factor that influence attractiveness (Jones et al., 2004). Facial symmetry and averageness also affect attractiveness (Grammer and Thornhill, 1994; Jones et al., 2001). However, skin color is more susceptible to change compared with facial symmetry and averageness, which may make it a useful indicator of health status and attractiveness (Little et al., 2011).

In addition to apparent health, the relationship between skin color and attractiveness is influenced by geographic and cultural backgrounds. In Asian countries, skin brightness is an important indicator of beauty. For example, in Japan, skin brightness is associated with beauty, and brightening cosmetics are favored (Ashikari, 2005; Li et al., 2008). Meanwhile, tanning is prevalent among Caucasians in the West, and darker skin is preferred (Melia and Bulman, 1995). It has been suggested that Chinese people prefer increased brightness and decreased yellowness compared to Caucasians, who prefer increased redness more than the Chinese (Han et al., 2018). In contrast, Malaysian Chinese people reportedly chose increased yellowing over increased redness to indicate a healthier appearance (Tan and Stephen, 2019). In a study using Japanese, Chinese, Thai, and Caucasian facial images, He et al. (2024) reported that East Asian cultures rated reddish skin tones positively, while Caucasians rated yellowish skin tones positively. Thus, skin color preferences vary according to geographic conditions and cultural differences.

The relationship between facial features and impressions has been studied experimentally using facial images (Jaeger et al., 2018; Samson et al., 2010). These studies were conducted in laboratories using print media or computer displays, and evaluated under appropriately controlled lighting conditions. Uniform visual conditions of stimuli presentation reduce noise during the evaluation. However, recently, online surveys have been used to evaluate facial images (Marini et al., 2022; Takehara et al., 2023). In online surveys, the experimenter has no control over the participant’s monitor or lighting conditions, which may lead to larger errors in stimulus evaluation. However, on comparing the results of laboratory experiments and online surveys, it was found that a sufficient sample size could effectively cancel out evaluation noise (Hirao et al., 2021). Online surveys do not require experimenters to prepare special laboratory equipment or travel and can be used to recruit a large number of participants from a wide range of geographic areas. In the present study, online surveys were used to recruit participants from different regions.

The purpose of this study was to determine the effects of intra-individual skin color changes on attractiveness using averaged male and female facial images, as well as to identify differences in geographic and cultural backgrounds. We qualitatively compared the relationship between attractiveness and skin color changes in countries with both near and distant geographic and cultural backgrounds. The study was conducted in Japan and China, which are relatively close geographically and culturally, as well as Italy, which is relatively different. Japan and China, located in East Asia, reportedly share a preference for white skin color (Li et al., 2008). However, while China is influenced by Western culture, the preference for whiteness in Japan may be based on their racial identity, which suggests that the cultural backgrounds may differ (Ashikari, 2005; Li et al., 2008). Italy, located in Europe, is geographically distant from the other two countries, and Caucasians tend to prefer darker skin (Caisey et al., 2006; Melia and Bulman, 1995). As a reference value for the short-term intra-individual changes in skin tone, we used the skin tone change value 1 h after applying a mild psychological stress load, in a previous experiment conducted by the authors (Koizumi et al., 2023, data not shown). The skin color change reflected blood retention and deoxygenation caused by capillary constriction due to psychological stress, and the skin color changed through decreasing L* and b* and increasing a* values. The CIELab color space allows for color representation based on human vision (Martinkauppi, 2002). The Lab* color space is a colorimetric system where L* represents lightness and a* and b* indicate the position on the red-green and yellow-blue axes, respectively, which provides a comprehensive measure of skin color attributes. A decrease in L* corresponds to a darker skin tone, an increase in a* indicates greater redness, and a decrease in b* is associated with stronger blue components. Although Koizumi et al. (2023) conducted their study on Japanese males only, the same skin color change rates were used for the male and female Japanese, Chinese, and Caucasian facial images in this study. By applying these skin color change rates, facial images that reproduced skin color changes within individuals were created and used in online surveys in each country. In addition, to understand the effect of each L* a* b* value on attractiveness, we created images in which only brightness was changed, only redness was changed, and both brightness and redness were changed, assuming that cosmetics are used to control skin color. Clarifying the effects of skin color changes on attractiveness will advance the development of skincare and makeup products that improve personal attractiveness.

## 2 Materials and methods

### 2.1 Participants

Surveys were conducted in Japan, China, and Italy to investigate the impact of skin color changes on attractiveness. In the Japanese study, 1,200 healthy individuals living in Japan (600 men and 600 women; mean age: 40.0 years; SD: 11.2) participated in the survey. In the Chinese study, 1,518 healthy individuals who lived in China (717 men and 801 women; mean age: 38.9 years; SD: 10.4) participated in the survey. In the Italian study, 1,200 healthy individuals who lived in Italy (600 men and 600 women; mean age: 39.5 years; SD: 11.5) participated in the survey. This number was considered sufficient to detect a 10% difference in selection rate in a chi-squared test with *α* = 0.05 and 1 − *β* = 0.8. None of the participants indicated optical disorders via self-report before the experiment, and all of them owned a personal computer or tablet, excluding mobile phones with small displays. All the participants provided informed consent. The Research Ethics Committee of the Shiseido Global Innovation Center approved this study and all methods followed the approved guidelines.

### 2.2 Stimuli

**Figure 1:**
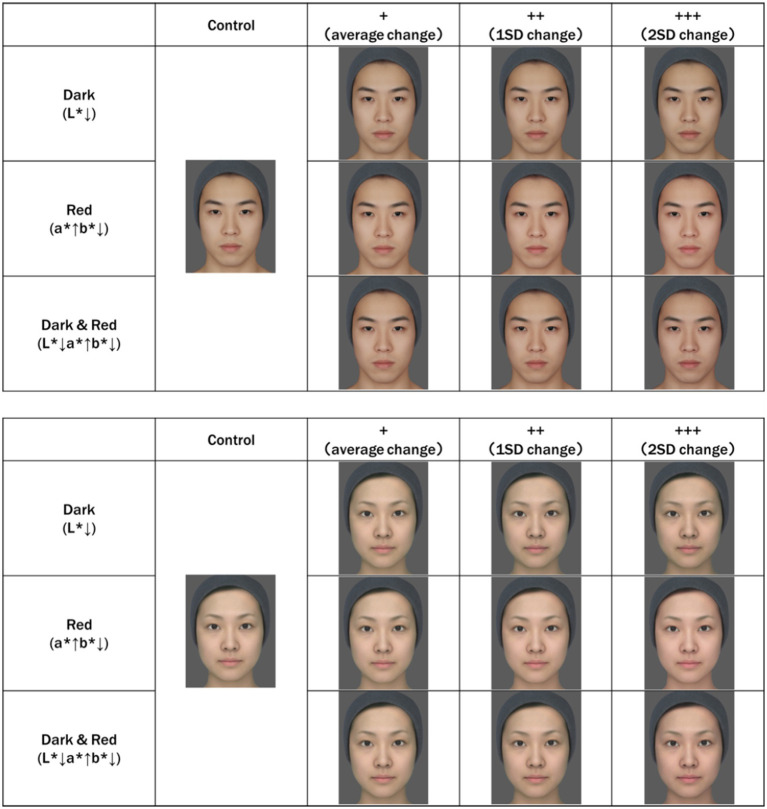
Average Japanese facial images. The original average face was created by averaging eight facial images of Japanese men and women in their 20s, obtained in a previous study (unpublished). The shape of the facial image was averaged based on the marker point coordinates of the acquired facial image. Skin color was adjusted based on skin color data for the same age and sex, as in previous studies. “Dark” is a facial image in which only L* is reduced from the original facial image. “Red” is a facial image in which a* is increased and b* is decreased from the original facial image. Dark&Red is a facial image in which L* and b* are decreased and a* is increased from the original facial image. In addition, to examine the effect of the degree of skin color change, we created images with an average change (+), 1SD change (++), and 2SD change (+++). The values (data not shown) obtained in a previous study were used (Koizumi et al., 2023). Created with FantaMorph.

The Japanese survey used 20 averaged computer generated (CG) Japanese facial photographs as visual stimuli. We also used two original average faces (a man and woman in their 20s) created in a previous study (Hirao et al., 2021). Facial photographs to create the average face were taken via a digital camera in an environment where the lighting conditions were kept constant. The hair was secured to avoid covering the face, and the digital camera was positioned directly in front of the face with the eyes open. Images were saved as 1,272 × 1,598 pixels and the average face was created via Photoshop CS4 (version 11; Adobe Inc., 2008). The shape of the facial image was averaged based on the marker point coordinates of the acquired facial image. Skin color was adjusted based on skin color data for the same age and sex, as in previous studies. The same procedure was used to create the original average faces of women in their 20s. To investigate the influence of skin color changes on facial impressions, images were created in which only L* decreased from the original image (Dark), images in which a* increased and b* decreased (Red), and images in which L* and b* decreased and a* increased (Dark & Red). The values (data not shown) obtained in a previous study were used (Koizumi et al., 2023). The skin color change value was the average increase/decrease rate and standard deviation (SD) of cheek L* a* b* values of 13 Japanese men 1 h after mild psychological stress loading, and was used as the reference value for skin color changes that occurred within the same person within a few hours. In addition, to examine the effect of the degree of skin color change, we created images with an average change (+), 1SD change (++) and 2SD change (+++). Skin color was manipulated using the average increase/decrease rate and SD so that L* and b* decreased and a* increased. Table 1 lists the skin color change rates. Ten images were created: one original image, three dark images (+, ++, +++), three red images (+, ++, +++), and three Dark & Red images (+, ++, ++). These were created for each age group and sex, resulting in 20 total images. These images are shown in Figure 1.

**Table 1:** The skin color change rates.

Changed color items	Control	+ (average change)	++ (1SD change)	+++ (2SD change)
L*	0%	−0.9%	−2.5%	−4.1%
a*	0%	+5.3%	+17.6%	+29.9%
b*	0%	−0.5%	−4.7%	−8.9%

**Figure 2:**
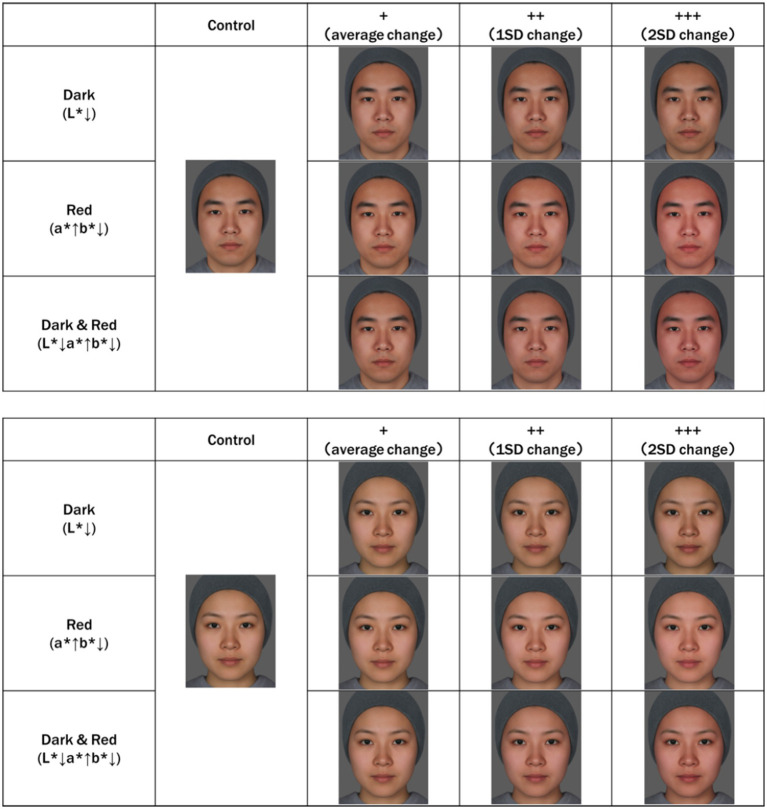
Average Chinese facial images. The original average face was created by averaging eight facial images of Chinese men and women in their 20s, obtained in a previous study (unpublished). The shape of the facial image was averaged based on the marker point coordinates of the acquired facial image. Skin color was adjusted based on skin color data for the same age and sex, as in previous studies. “Dark” is a facial image in which only L* is reduced from the original facial image. “Red” is a facial image in which a* is increased and b* is decreased from the original facial image. Dark&Red is a facial image in which L* and b* are decreased and a* is increased from the original facial image. In addition, to examine the effect of the degree of skin color change, we created images with an average change (+), 1SD change (++), and 2SD change (+++). The values (data not shown) obtained in a previous study were used (Koizumi et al., 2023). Created with FantaMorph.

**Figure 3:**
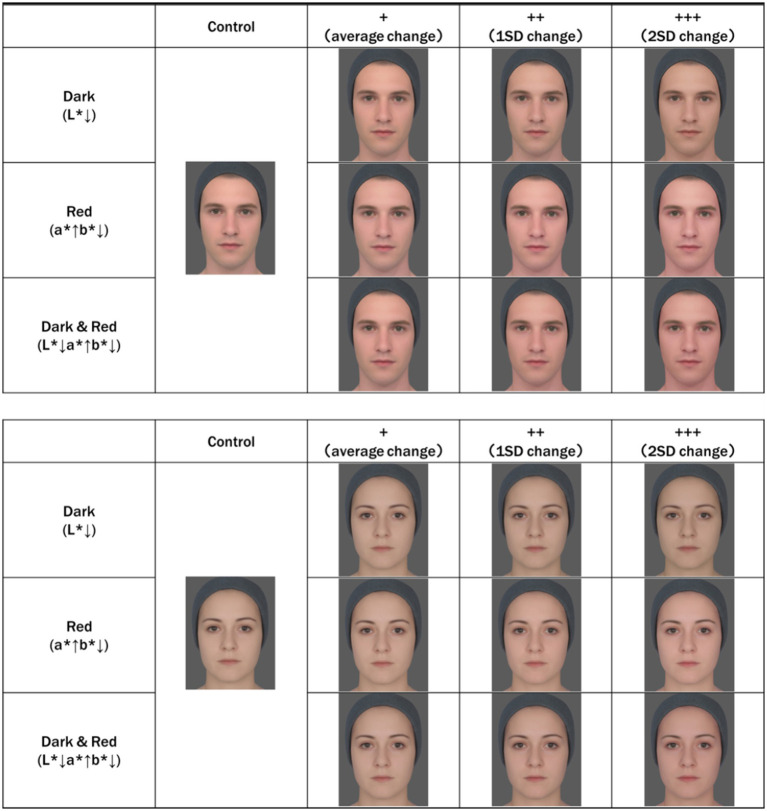
Average Caucasian facial images. The original average face was created by averaging eight facial images of Caucasian men and women in their 20s, obtained in a previous study (unpublished). The shape of the facial image was averaged based on the marker point coordinates of the acquired facial image. Skin color was adjusted based on skin color data for the same age and sex, as in previous studies. “Dark” is a facial image in which only L* is reduced from the original facial image. “Red” is a facial image in which a* is increased and b* is decreased from the original facial image. Dark&Red is a facial image in which L* and b* are decreased and a* is increased from the original facial image. In addition, to examine the effect of the degree of skin color change, we created images with an average change (+), 1SD change (++), and 2SD change (+++). The values (data not shown) obtained in a previous study were used (Koizumi et al., 2023). Created with FantaMorph.

The same procedure was used to create 20 Chinese and Caucasian face images for the Chinese and Italian surveys. The original average face was created by averaging eight images of Chinese or Caucasian men in their 20s obtained in a previous study (unpublished). The average Caucasian male and female faces were created from facial images of eight Caucasian men recruited in France and eight Caucasian women recruited in Italy, respectively. Skin color was adjusted based on skin color data for the same age and sex, as obtained in a previous study (unpublished data). The same procedure was used to create the original average face of women in their 20s. Ten images were created for both sexes by adjusting L*, a*, and b* in the same skin color change rates as in the Japanese study. The resulting Chinese and Caucasian facial images are shown in Figures 2, 3, respectively.

### 2.3 Questionnaire

The questionnaire included two perceptual items that used bipolar scales (bright–dark and red–yellow) and “attractiveness” impression of the face measured using a monopole scale. Perceptual questions were used to confirm how the participants perceived the skin color of the facial images. All questionnaire items used a five-point Likert scale, with scores ranging from −2 to +2. For example, the bright–dark dipole scale appeared as follows: +2, “match to bright”; +1, “a slight match to bright”; 0, neutral; −1, “match to dark”; and −2, “a slight match to dark.” The monopole scale categories appeared as: +2, “I think so very much”; +1, “I think so”; 0, neutral; −1, “I do not think so”; and −2, “I do not think so very much.”

### 2.4 Procedure

The participants were divided into two groups: one evaluated facial images of men in their 20s and the other evaluated facial images of women in their 20s. The participants viewed the stimuli on their personal computers or tablets and completed the questionnaire at home. At the beginning of the questionnaire, the participants were requested to select the terminal that they were using to respond to the questionnaire. Participants who chose any option other than desktops, laptops, or tablets were excluded. The facial images were evaluated individually. One facial image was displayed at the center of the monitor, and two perceptual questions were presented below the image. When the participant answered the questions and clicked, the facial image changed. This process was repeated for 10 images. Afterwards, a facial image was displayed at the center of the monitor, and questions about attractiveness were displayed below. The facial images were changed each time the question was answered, and the process was repeated for 10 images. Images and questions were switched at the participants’ pace, and there was no time limit for responses. Order of appearance of the images was randomized. The survey in Japan was conducted by Nippon Information Inc., while the surveys in China and Italy were conducted by Glocal Insight Co., Ltd. Each survey’s interface was adjusted to ensure a consistent format.

### 2.5 Statistical analysis

The proportion of participants who selected “match to bright (or red)” and “a slight match to bright (or red)” for the perception question was calculated (impression score). The impression score for the attractiveness question was calculated as the proportion of participants who selected “I think so very much” and “I think so.” A chi-squared test was used to determine whether there was a significant difference from the original average face. The null hypothesis was that there would be no difference in the ratio of the number of respondents to the questions for the original facial images. *p*-values were corrected using the Bonferroni method. The significance level *α* was set at α = 0.05.

## 3 Results

### 3.1 The Japanese study

Table 2 shows the impression scores from the perception questionnaire for Japanese facial images. For the item “Looks bright,” impression scores of the Dark (+, ++, and +++) and Dark&Red (+, ++, and +++) images of male and female faces significantly decreased compared to the original facial image. For men in their 20s, in the Red face, impression scores of “Looks bright” decreased even though L* was not changed [Red+: χ^2^ (1) = 12.07, *p* = 0.009; Red++: χ^2^ (1) = 12.71, *p* = 0.007; Red+++: χ^2^ (1) = 23.48, *p* < 0.001]. In contrast, for women in their 20s, in the Red face, impression scores of “Looks bright” increased [Red++: χ^2^ (1) = 59.63, *p* < 0.001, Red+++: χ^2^ (1) = 63.50, *p* < 0.001].

**Table 2:** The impression score on the perception questionnaire for Japanese facial images.

Facial image	Men in their 20s		Women in their 20s	
	Looks bright	Looks red	Looks bright	Looks red
Original	72.5%	9.8%	55.5%	14.2%
Dark+	61.7%*	10.0%	32.7%*	10.3%
Dark++	53.3%*	12.2%	23.5%*	14.5%
Dark+++	43.8%*	9.8%	12.7%*	15.8%
Red+	66.2%*	17.7%*	57.7%	13.7%
Red++	66.0%*	42.7%*	71.2%*	36.0%*
Red+++	63.7%*	66.5%*	71.7%*	70.0%*
Dark & Red+	59.5%*	23.7%*	38.8%*	16.7%
Dark & Red++	47.0%*	32.3%*	35.5%*	24.7%*
Dark & Red+++	36.2%*	43.7%*	34.2%*	62.2%*

* indicates that the *p-*value of the Bonferroni-corrected chi-square test for the original was less than 5%.

For the item “Looks red,” impression scores of male Red and Dark&Red faces increased [20s male Red+: χ^2^ (1) = 41.52, *p* < 0.001; 20s male Red++: χ^2^ (1) = 729.51, *p* < 0.001; 20s male Red+++: χ^2^ (1) = 2173.00, *p* < 0.001; 20s male Dark&Red+: χ^2^ (1) = 129.50, *p* < 0.001; 20s male Dark&Red++: χ^2^ (1) = 342.59, *p* < 0.001; 20s male Dark&Red+++: χ^2^ (1) = 774.63, *p* < 0.001]. For women in their 20s, impression scores of Red++, Red+++, Dark&Red++, and Dark&Red+++ significantly increased [Red++: χ^2^ (1) = 235.22, *p* < 0.001; Red+++: χ^2^ (1) = 1538.21, *p* < 0.001; Dark&Red++: χ^2^ (1) = 54.40, *p* < 0.001; Dark&Red+++: χ^2^ (1) = 1136.87, *p* < 0.001]. No increase or decrease in redness was observed in the facial images in which a* did not change.

Table 3 shows the impression scores for the attractiveness of Japanese facial images. For male faces, the impression score of attractiveness of the Dark+++ face significantly decreased (χ^2^ (1) = 9.69, *p* = 0.017). In comparison, impression scores of attractiveness of male Red+ and Red++ faces significantly increased [Red+: χ^2^ (1) = 10.25, *p* = 0.012; Red++: χ^2^ (1) = 12.71, *p* = 0.007; Red+++: χ^2^ (1) = 10.25, *p* = 0.012]. For female faces, impression scores of Dark and Dark&Red faces significantly decreased [Dark+: χ^2^ (1) = 58.89, *p* < 0.001; Dark++: χ^2^ (1) = 100.70, *p* < 0.001; Dark+++: χ^2^ (1) = 143.39, *p* < 0.001; Dark&Red+: χ^2^ (1) = 9.73, *p* = 0.016; Dark&Red++: χ^2^ (1) = 13.77, *p* = 0.002; Dark&Red+++: χ^2^ (1) = 20.74, *p* < 0.001]. In contrast, impression scores of female Red++ and Red+++ faces significantly increased [Red++: χ^2^ (1) = 95.69, *p* < 0.001; Red+++: χ^2^ (1) = 45.51, *p* < 0.001].

**Table 3:** The impression score for attractiveness of Japanese facial images.

Facial image	Men in their 20s	Women in their 20s
Original	30.2%	37.5%
Dark+	28.5%	22.3%*
Dark++	29.7%	17.7%*
Dark+++	24.3%*	13.8%*
Red+	36.2%*	39.8%
Red++	36.2%*	56.8%*
Red+++	34.5%	50.8%*
Dark & Red+	32.5%	31.3%*
Dark & Red++	30.8%	30.2%*
Dark & Red+++	26.0%	28.5%*

* indicates that the *p*-value of the Bonferroni-corrected chi-square test for the original was less than 5%.

### 3.2 The Chinese study

Table 4 shows the impression scores of the perception questionnaires for Chinese facial images. For the category “Looks bright,” impression scores of the Dark++, Dark+++, Dark&Red+, and Dark&Red+++ facial images of men in their 20s significantly decreased compared to the original facial image [Dark++: χ^2^ (1) = 63.43, *p* < 0.001; Dark+++: χ^2^ (1) = 90.77, *p* < 0.001; Dark&Red+++: χ^2^ (1) = 21.99, *p* < 0.001]. For the faces of women in their 20s, impression scores of Dark+, Dark++, Dark+++, and Dark&Red+++ faces decreased compared to the original facial image [Dark+: χ^2^ (1) = 9.31, *p* = 0.041; Dark++: χ^2^ (1) = 31.30, *p* < 0.001; Dark+++: χ^2^ (1) = 76.02, *p* < 0.001; Dark&Red+++: χ^2^ (1) = 41.82, *p* < 0.001]. Meanwhile, impression scores of female Red++ and Red+++ faces increased [Red++: χ^2^ (1) = 13.20, *p* = 0.005; Red+++: χ^2^ (1) = 27.37, *p* < 0.001]. For the question “Looks red,” impression scores increased for all facial images with changes in a* compared to the original facial image. Furthermore, impression scores increased for some facial images where a* did not change [20s male Dark+: χ^2^ (1) = 10.36, *p* = 0.023; 20s male Dark++: χ^2^ (1) = 15.65, *p* = 0.001; 20s female Dark+++: χ^2^ (1) = 12.58, *p* = 0.007].

**Table 4:** The impression score on the perception questionnaire for Chinese facial images.

Facial image	Men in their 20s		Women in their 20s	
	Looks bright	Looks red	Looks bright	Looks red
Original	42.9%	18.6%	41.4%	14.6%
Dark+	42.7%	23.2%*	36.0%*	15.1%
Dark++	28.4%*	25.5%*	31.5%*	16.0%
Dark+++	25.5%*	18.9%	26.0%*	19.1%*
Red+	39.3%	24.0%*	46.0%	18.4%*
Red++	41.7%	61.7%*	47.8%*	53.6%*
Red+++	42.2%	79.6%*	50.6%*	70.8%*
Dark & Red+	33.0%*	25.1%*	36.6%	24.3%*
Dark & Red++	37.4%	59.0%*	37.4%	64.3%*
Dark & Red+++	34.3%*	76.8%*	30.0%*	74.5%*

* indicates that the *p*-value of the Bonferroni-corrected chi-square test for the original was less than 5%.

Table 5 lists the impression scores of the Chinese facial images. In Dark++, Dark+++, Red+++, and all Dark&Red facial images of men in their 20s, impression scores of attractiveness significantly decreased [Dark++: χ^2^ (1) = 30.56, *p* < 0.001; Dark+++: χ^2^ (1) = 49.04, *p* < 0.001; Red+++: χ^2^ (1) = 69.38, *p* < 0.001; Dark&Red+: χ^2^ (1) = 24.39, *p* < 0.001; Dark&Red++: χ^2^ (1) = 19.56, *p* < 0.001; Dark&Red+++: χ^2^ (1) = 104.98, *p* < 0.001]. For women in their 20s, impression scores significantly decreased for Dark++, Dark+++, Dark&Red++, and Dark&Red+++ facial images (Dark++: χ^2^ (1) = 9.05, *p* = 0.024; Dark+++: χ^2^ (1) = 32.84, *p* < 0.001; Dark&Red++: χ^2^ (1) = 13.35, *p* = 0.002; Dark&Red+++: χ^2^ (1) = 59.85, *p* < 0.001).

**Table 5:** The impression score for attractiveness of Chinese facial images.

Facial image	Men in their 20s	Women in their 20s
Original	51.7%	52.1%
Dark+	51.0%	47.5%
Dark++	41.5%*	46.7%*
Dark+++	38.8%*	41.9%*
Red+	47.2%	50.7%
Red++	48.4%	52.0%
Red+++	36.4%*	53.4%
Dark & Red+	42.6%*	49.9%
Dark & Red++	43.6%*	45.6%*
Dark & Red+++	32.8%*	38.3%*

* indicates that the *p*-value of the Bonferroni-corrected chi-square test for the original was less than 5%.

### 3.3 The Italian study

Table 6 shows the impression scores on the perception questionnaire for the Caucasian facial images. For the category “Looks bright,” impression scores of the Dark++ and Dark+++ male and female faces significantly decreased compared to the original facial image [20s male Dark++: χ^2^ (1) = 38.92, *p* < 0.001; 20s male Dark+++: χ^2^ (1) = 80.06, *p* < 0.001; 20s female Dark++: χ^2^ (1) = 38.76, *p* < 0.001; 20s female Dark+++: χ^2^ (1) = 86.43, *p* < 0.001]. For men in their 20s, impression scores for the Dark&Red face decreased [Dark&Red+: χ^2^ (1) = 13.46, *p* = 0.005; Dark&Red++: χ^2^(1) = 39.95, *p* < 0.001; Dark&Red+++: χ^2^ (1) = 38.92, *p* < 0.001]. For the male Red+++ face, impression scores of “Looks bright” decreased even though L* was not changed [χ^2^ (1) = 11.87, *p* = 0.010]. For female faces, impression scores significantly decreased for Dark&Red+ and Dark&Red++ faces [Dark&Red+: χ^2^ (1) = 16.54, *p* = 0.001; Dark&Red++: χ^2^(1) = 13.34, *p* = 0.005]. In contrast, for the Red+++ face of women in their 20s, impression scores of “Looks bright” increased [χ^2^ (1) = 39.80, *p* < 0.001].

**Table 6:** The impression score on the perception questionnaire for Caucasian facial images.

Facial image	Men in their 20s		Women in their 20s	
	Looks bright	Looks red	Looks bright	Looks red
Original	44.8%	39.3%	41.0%	7.8%
Dark+	45.7%	22.2%*	39.5%	13.3%*
Dark++	32.2%*	14.8%*	28.5%*	15.7%*
Dark+++	26.7%*	8.5%*	22.3%*	12.0%*
Red+	45.2%	47.2%*	39.8%	32.5%*
Red++	39.0%	81.3%*	39.5%	71.3%*
Red+++	37.8%*	83.8%*	53.7%*	82.5%*
Dark & Red+	37.5%*	49.5%*	32.8%*	38.0%*
Dark & Red++	32.0%*	70.7%*	33.7%*	69.7%*
Dark & Red+++	32.2%*	86.5%*	42.3%	87.2%*

* indicates that the *p*-value of the Bonferroni-corrected chi-square test for the original was less than 5%.

For the category “Looks red,” impression scores of all Red and Dark&Red faces significantly increased compared to the original faces. For the male face, impression scores of “Looks red” in Dark faces significantly decreased [Dark+: χ^2^ (1) = 74.10, *p* < 0.001; Dark++: χ^2^ (1) = 150.93, *p* < 0.001; Dark+++: χ^2^ (1) = 239.05, *p* < 0.001]. In comparison, for female faces, impression scores of “Looks red” in Dark faces increased [Dark+: χ^2^ (1) = 25.14, *p* < 0.001; Dark++: χ^2^ (1) = 50.99, *p* < 0.001; Dark+++: χ^2^ (1) = 14.43, *p* = 0.003].

Table 7 shows the impression scores for attractiveness of the Caucasian facial images. For men in their 20s, the impression score of attractiveness in the Red+++ and Dark&Red+++ faces significantly decreased compared to the original face [Red+++: χ^2^ (1) = 9.79, *p* = 0.016; Dark&Red+++: χ^2^ (1) = 11.40, *p* = 0.007]. However, impression scores of attractiveness in the female Red++ and Red+++ faces significantly increased [Red++: χ^2^ (1) = 11.32, p = 0.007; Red+++: χ^2^ (1) = 13.08, *p* = 0.003].

**Table 7:** The impression score for attractiveness of Caucasian facial images.

Facial image	Men in their 20s	Women in their 20s
Original	43.5%	38.0%
Dark+	43.3%	41.3%
Dark++	41.2%	36.0%
Dark+++	39.0%	35.2%
Red+	43.3%	42.0%
Red++	44.0%	44.7%*
Red+++	37.2%*	45.2%*
Dark & Red+	41.8%	42.0%
Dark & Red++	42.3%	43.3%
Dark & Red+++	36.7%*	40.5%

* indicates that the *p*-value of the Bonferroni-corrected chi-square test for the original was less than 5%.

## 4 Discussion

This study aimed to elucidate the impact of individual skin color changes on attractiveness via average facial images of both men and women and examine the influence of geographical and cultural backgrounds. To qualitatively compare the relationship between attractiveness and changes in skin color in countries with similar and different geographical and cultural backgrounds, experiments were conducted in Japan, China, and Italy. To examine the effect of skin color changes on facial impressions, images were derived from the original by decreasing only L* (Dark), increasing a* and decreasing b* (Red), and decreasing L* and b* while increasing a* (Dark & Red). Additionally, to investigate the impact of the degree of skin color change, images with average (+), 1SD (++), and 2SD changes (+++) were created. Questions regarding perception and attractiveness were designed to examine how changes in skin color were perceived. Investigating the impact of individual skin color changes on attractiveness and differences owing to geographical and cultural backgrounds is crucial for the development of global skincare and makeup products.

### 4.1 Skin color perception

Perception questions identified how changes in skin color were perceived. In all the surveys, a decrease in apparent brightness due to a decrease in L* and an increase in apparent redness due to an increase in a* and decrease in b* were observed. In Red faces, an increase in apparent brightness was observed in female faces in all the studies. An increase in a* increased apparent brightness in Japanese female faces (Yoshikawa et al., 2012). Our results supported those of previous studies and revealed that this illusion effect occurred in Japanese and also Chinese and Caucasian faces.

In contrast, changes in apparent redness due to a decrease in L* and apparent brightness due to increases in a* and decreases in b* were also observed. In the Japanese study, no change in apparent redness with decreasing L* was observed. Meanwhile, in the Chinese study, appearance of redness increased with decreasing L* only in the male face. In the Italian study, a decrease in L* decreased and increased the appearance of redness on male and females faces, respectively. Perception of skin color in human faces was influenced by the skin colors encountered in daily life (Shimakura and Sakata, 2022). Tanning influences skin color changes in daily life; however, the way skin color changes due to tanning also differs (Fitzpatrick, 1988). Asians, such as Japanese and Chinese individuals, tend to become relatively darker after they tan (Chan et al., 2019; Liu et al., 2006). Conversely, Caucasians are more likely to develop a reddish hue when tanned (Gupta and Sharma, 2019). These differences in common skin color changes may have influenced the perception of skin color. Additionally, differences between East Asian and Western Caucasian cultures influenced facial recognition processes (Blais et al., 2008). Hence, perception of skin color may be influenced by physiological characteristics and cultural backgrounds. However, to understand whether skin color perception varies based on geographical and cultural backgrounds, further controlled studies are warranted.

### 4.2 Influence of skin color on attractiveness

The impression questions examined the impact of skin color change on attractiveness. In the Japanese and Chinese survey, attractiveness decreased for Dark faces of both sexes. Conversely, no change in attractiveness was observed for Dark faces in the Italian survey. Skin brightness is an important indicator of beauty in Asian countries, including China and Japan (Ashikari, 2005; Li et al., 2008). Reduced attractiveness due to reduced brightness in Chinese and Japanese faces in this study supported the results of previous studies.

In contrast, Western Caucasians tend to favor tanning (Melia and Bulman, 1995). Indeed, darker skin is more attractive than lighter skin on Caucasian female faces (Fink et al., 2001). However, research revealed that an increase in L* increased apparent health (Stephen et al., 2011). In this study, no change in attractiveness was observed for faces with a decrease in L* alone. This finding was not consistent with those of previous studies. Skin color changes reproduced in this study referred to the changes in skin color that could occur within the same day: L* was reduced by a maximum of 3 units, a smaller change than that in Stephen et al.’s (2011) study (−5.2 units). Thus, our findings may differ from those of previous reports owing to the relatively small color changes. Furthermore, the effect of L* on facial attractiveness was considered to be larger in Japan and China than in Italy, as the decrease in L* caused a decrease in attractiveness in Japan and China. These results indicate that the effects of L* reduction on facial attractiveness are similar in Japan and China, which are in Asia, as reported. Conversely, in Italy, which is geographically and culturally distant from Japan and China, the effect of L* reduction on facial attractiveness may be less significant.

The influence of the Red faces on attractiveness varied across different countries. Facial redness increases attractiveness (Stephen et al., 2009; Thorstenson et al., 2017). In fact, for Japanese faces, attractiveness increased for Red faces of both sexes. In contrast, attractiveness tended to decrease for Chinese faces, except for female faces. Furthermore, Caucasian faces exhibited an increase in attractiveness for Red faces of females, whereas attractiveness decreased for male faces. In male faces, redness increased attractiveness and also aggression and dominance (Stephen et al., 2012), and a gender bias existed regarding dominance (Oh et al., 2020). Opposite results of changes in attractiveness due to redness in Caucasian male and female faces may have been influenced by gender biases regarding dominance and aggression. Furthermore, gender bias may have also influenced the decrease in attractiveness observed in the Chinese male Red faces. For Japanese faces, attractiveness increased for Red faces of both sexes. Different effects of facial redness on attractiveness compared with Chinese faces suggested that the effects of facial color on attractiveness may differ even between regions with close geographic and cultural backgrounds. In fact, the Japanese preferred reddish faces (He et al., 2024). Furthermore, this trend was more pronounced compared with that of Chinese individuals. Thus, the results support regional differences in attractiveness associated with changes in skin color. However, detailed studies should determine the effect of redness on attractiveness, as the original facial shape and skin color also differ.

This study examined the effects of skin color changes on attractiveness of the same person in Japan, China, and Italy. Consequently, it was revealed that changes in skin color within the same individual affected attractiveness in all the surveys. Furthermore, changes in attractiveness varied by country. Influence of skin brightness on attractiveness demonstrated similar trends in Japan and China; however, different trends were observed regarding skin redness, even between geographically close countries, such as Japan and China. Differences in the effects of these skin tones on attractiveness may be influenced by cultural backgrounds. Turner (2012) proposed the concept of “social skin,” stating that skin serves as a social boundary. Yamamoto et al. (2002) reported that preferred skin tones were influenced by cultural factors based on surveys conducted in Japan and South Korea. In Japan, the phenomenon of skin whitening has become a social trend, as evidenced by the prevalence of advertisements for whitening cosmetics (Ashikari, 2005). Furthermore, differences in women’s physical preferences and men’s masculinity across cultures suggest that gender bias may vary between cultures (Swami and Tovée, 2005; DeBruine et al., 2010). Differences in the impact of skin tone on attractiveness observed in this study could be owing to a complex interplay of social and cultural backgrounds. Future studies should examine how the influence of skin color on attractiveness is shaped by cultural backgrounds. These findings indicate the importance of global cosmetics development based on the fact that preferred skin color changes vary according to geographic and cultural backgrounds and sex.

### 4.3 Limitations

This study has several limitations. From the perspective of personal information protection and ease of control, average faces were used; however, morphed faces lose individual features, such as expressions (Korshunov and Ebrahimi, 2013). To investigate the impact of skin color on attractiveness in the real world, considering elements, such as expressions and morphology, is necessary. The facial images used in this study had original Lab* values that differed across countries. Therefore, we cannot exclude the possibility that differences in the initial skin color values influenced the results. Additionally, regarding the amount of skin color change, we used values from previous studies conducted on Japanese individuals for standardization; however, physiological verification is required to determine whether the same changes occur in faces from different countries. Furthermore, since this survey used different stimuli and participants, quantitative comparisons could not be made. Moreover, the survey was conducted online; hence, visual conditions, such as monitors and lighting, may have varied across the studies. Future controlled research should conduct quantitative comparisons. This study utilized a 5-point Likert scale to evaluate attractiveness. However, biases in response styles, such as acquiescence response and mid-point response styles, may vary across cultures (Van Vaerenbergh and Thomas, 2013). Furthermore, the perception of attractiveness involves multiple factors, such as perceived healthiness, and personality traits, such as aggressiveness, which may not be adequately evaluated via a single-dimensional scale, such as the Likert scale (Stephen et al., 2012; Thorstenson et al., 2017). Future studies should incorporate evaluation methods that consider these biases and the complexity of attractiveness. In this study, the survey that used images of Caucasian faces was conducted in Italy; however, the perception of attractiveness may differ among different European populations. A study examined the roles of skin color, hair color, and body weight in the perception of female attractiveness across eight countries and found that even among the same Western countries, evaluations of attractive skin tones varied (Swami et al., 2008). Future research should explore the impact of skin color on attractiveness among Caucasians with different cultural backgrounds.

## 5 Conclusion

This study examined how skin color changes that can occur within individuals affect attractiveness in Japanese, Chinese, and Caucasian faces, using averaged male and female facial images. In each country, we found that intra-individual changes in skin color affected facial attractiveness. The effects of skin color change on attractiveness differed not only between Japan and Italy, which have different geographic and cultural backgrounds, but also between Japan and China, which are relatively close. Furthermore, the results indicated a gender bias in the perception of facial attractiveness, even within the same country. These results reveal that the impact of intra-individual skin color variation on attractiveness varies by geographic and cultural backgrounds and sex, and will advance the development of global skincare and makeup products that improve individual attractiveness.

## Data Availability

The datasets presented in this article are not readily available because owing to confidentiality agreements with the participants. The data used in this study are available only at the Shiseido Global Innovation Center. Requests to access the datasets should be directed to koyo.koizumi@shiseido.com.
